# *Staphylococcus aureus*–associated Skin and Soft Tissue Infections in Ambulatory Care

**DOI:** 10.3201/eid1211.060190

**Published:** 2006-11

**Authors:** Linda F. McCaig, L. Clifford McDonald, Sanjay Mandal, Daniel B. Jernigan

**Affiliations:** * Centers for Disease Control and Prevention, Hyattsville, Maryland, USA;; † Centers for Disease Control and Prevention, Atlanta, Georgia, USA

**Keywords:** Skin and soft tissue infections, Staphylococcus aureus, ambulatory care, research

## Abstract

To describe the number and treatment of skin and soft tissue infections likely caused by *Staphylococcus aureus* in the United States, we analyzed data from the 1992–1994 and 2001–2003 National Ambulatory Medical Care Surveys and National Hospital Ambulatory Medical Care Surveys. Each year, data were reported by an average of 1,400 physicians, 230 outpatient departments, and 390 emergency departments for 30,000, 33,000, and 34,000 visits, respectively. During 2001–2003, the number of annual ambulatory care visits for skin and soft tissue infections was 11.6 million; the visit rate was 410.7 per 10,000 persons. During the study period, rates of overall and physician office visits did not differ; however, rates of visits to outpatient and emergency departments increased by 59% and 31%, respectively. This increase may reflect the emergence of community-acquired methicillin-resistant S. aureus infections.

## Abstract 2

The rise in visits to outpatient and emergency departments for skin and soft tissue infections may reflect the emergence of community-associated methicillin-resistant *Staphylococcus aureus*.

Staphylococcus aureus is the almost-universal cause of furuncles, carbuncles, and skin abscesses and worldwide is the most commonly identified agent responsible for skin and soft tissue infections. S. aureus skin and soft tissue infections frequently begin as minor boils or abscesses and may progress to severe infections involving muscle or bone and may disseminate to the lungs or heart valves (i.e., endocarditis). Treatment of early infections consists of incising and draining the lesion, often accompanied by β-lactam antimicrobial drugs, which are also effective against β-hemolytic streptococci.

Strains resistant to β-lactam antimicrobial drugs, termed methicillin-resistant S. aureus (MRSA), were recognized from the 1960s through the 1990s as healthcare-associated (HA) pathogens (*1*). In the late 1990s, MRSA disease without established healthcare risk factors, called community-associated (CA)–MRSA, was increasingly reported in the literature (*2**,**3*). A study conducted in 2004 in emergency departments in 11 US cities found that MRSA was isolated from 59% of patients with skin and soft tissue infections (*4*). The biology of CA-MRSA appears to differ from that of HA-MRSA and CA–methicillin-susceptible S. aureus (MSSA), perhaps allowing CA-MRSA to cause disease other than that expected from MSSA (*5**–**8*). As HA-MRSA emerged, it likely did not merely replace HA-MSSA but led to an overall increase in S. aureus infections in healthcare settings (*9**–**11*).

Because most skin and soft tissue infections are treated in outpatient settings with empiric antimicrobial therapy, few studies have attempted to estimate the number of S. aureus skin and soft tissue infections, and none have evaluated the antimicrobial drugs prescribed for these conditions. Therefore, with regard to skin and soft tissue infections likely caused by S. aureus, we 1) estimated the number and rate of ambulatory care visits in the United States during 2 periods and examined any changes in these estimates between these periods; 2) described patient demographic characteristics; and 3) characterized antimicrobial and outpatient surgical therapy provided. Our results are based on a secondary data analysis of the 1992–1994 and 2001–2003 National Ambulatory Medical Care Surveys (NAMCS) and National Hospital Ambulatory Medical Care Surveys (NHAMCS).

## Methods

### Sample Design

NAMCS is a probability sample survey of office-based physicians in the United States, conducted by the National Center for Health Statistics of the Centers for Disease Control and Prevention (CDC). The US Bureau of the Census has been responsible for field operations and data collection since NAMCS became an annual survey in 1989. Sample design, sampling variance, and estimation procedures of the NAMCS have been described (*12*). NHAMCS is an annual probability sample survey of hospital outpatient departments and emergency departments in the United States, first conducted in 1992 by CDC's National Center for Health Statistics. The US Census Bureau is responsible for field operations and data collection. The plan and operation of NHAMCS have been described (*13*).

### Response Rates and Sample Size

From 1992 through 2003, response rates were 64%–73% for physician offices, 87%–91% for outpatient departments, and 90%–97% for emergency departments. The annual number of participating physicians was 1,000–1,800, outpatient departments 224–283, and emergency departments 364–425. The number of patient record forms completed each year by physician offices was 24,000–36,000, by outpatient departments 28,000–35,000, and by emergency departments 26,000–40,000. Estimates for skin and soft tissue infection visits are based on 3,374 sample records from 1992 through 1994 and 3,941 from 2001 through 2003.

### Data Collection and Coding

The same patient record form is used for the physician office and outpatient department settings, whereas the emergency department form differs slightly. The patient record form contains patient demographic data and information about the visit, including cause of injury, diagnosis, ambulatory surgical procedures (NAMCS and NHAMCS outpatient department), medications, and disposition. As many as 3 diagnoses are coded according to the International Classification of Diseases, Ninth Revision, Clinical Modification (ICD-9-CM) (*14*). During 2001–2003, 1–3 causes of injury were coded according to the Supplementary Classification of External Causes of Injury and Poisoning in the ICD-9-CM, and 1–2 ambulatory surgical procedures were coded to ICD-9-CM volume 3 (*14*). Cause of injury was not collected on the NAMCS and outpatient department patient record forms until 1995. From 1992 through 1994, 1–5 medications were recorded per visit; this number increased to 6 from 1995 through 2002 and 8 in 2003. Therapeutic classifications were based on the National Drug Code Directory (*15**,**16*). For this analysis, only 5 drugs per visit were included. A report describing the method and instruments used to collect and process drug information has been published (*17*).

### Definitions

Skin and soft tissue infections likely caused by S. aureus are defined as any diagnoses assigned the ICD-9-CM codes shown in Table 1. These infections were selected because of their likelihood of being caused by S. aureus as determined by the authors and as they appeared in a medical textbook (*18*). The ICD-9-CM code for Staphylococcus, 41.1, was not included because it is used as an additional code to identify the bacterial agent in diseases classified elsewhere. Few records with this code were found in NAMCS and NHAMCS data, most likely because cultures were either not performed or the results were not available at the time of the visit.

**Table 1:** Average annual percentage and rate of ambulatory care visits for selected skin and soft tissue infections, by diagnosis, United States*

Diagnosis	ICD-9-CM code	1992–1994				2001–2003			
		% visits†	95% CI	No. visits/10,000 persons/y	95% CI	% visits†	95% CI	No. visits/10,000 persons/y	95% CI
All visits		NA	NA	376.3	340.4–412.3	NA	NA	410.7	368.7–452.7
Inflammatory disease of breast	611.0	3.9	2.6–5.3	14.8	9.6–20.0	3.2	2.1–4.9	13.2	7.4–19.0
Carbuncle and furuncle	680	3.0	1.7–4.2	11.1	6.2–16.0	3.8	2.8–5.1	15.6	10.8–20.4
Cellulitis and abscess of finger and toe	681	9.4	7.7–11.2	35.5	28.0–43.1	9.7	7.6–12.3	39.8	29.6–50.1
Other cellulitis and abscess	682	46.2	42.6–49.9	174.0	150.4–197.7‡	53.2	49.1–57.2	218.4	188.7–248.1‡
Impetigo	684	14.3	11.5–17.2	53.9	42.2–65.6‡	8.9	6.9–11.6	36.7	26.4–47.0‡
Unspecified local infection of skin and subcutaneous tissue	686.9	9.6	7.2–11.9	36.1	26.9–45.3	8.0	6.3–10.1	32.7	24.8–40.6
Other specified diseases of hair and hair follicles	704.8	9.5	7.6–11.4	35.7	28.2–43.2	10.9	8.7–13.7	44.9	33.9–55.9
Hydradenitis	705.83	1.8	0.8–2.7	6.6	2.9–10.3	2.0	1.3–3.3	8.3	4.2–12.4
Other skin and soft tissue infections	§	3.4	1.7–5.1	12.9	6.6–19.2	1.9	1.1–3.2	7.8	3.5–12.1

*ICD-9-CM, International Classification of Diseases, Ninth Revision, Clinical Modification; CI, confidence interval; NA, not applicable. †Total exceeds 100% because >1 diagnosis may be reported per visit. ‡1992–1994 95% CI overlaps with 2001–03 95% CI, but p<0.05. §Includes ICD-9-CM codes 683 (acute lymphadenitis), 686.0 (pyoderma), 728.0 (myositis), 771.4 (omphalitis of the newborn), and 771.5 (neonatal infective mastitis).

The 1992–1994 denominators used in calculating the visit rates for age, sex, race, and geographic region are based on the Census Bureau estimates of the civilian, noninstitutional population of the United States as of July 1, 1992; July 1, 1993; and July 1, 1994, respectively. The 2001–2003 denominators are based on post–Census 2000 estimates of the civilian noninstitutional population of the United States. Population estimates of metropolitan statistical area status are based on data from the 2001, 2002, and 2003 National Health Interview Surveys, National Center for Health Statistics, adjusted to the US Census Bureau definition of core-based statistical areas. Denominators used to compute estimates of visit rates by expected source of payment were obtained from the 2001, 2002, and 2003 National Health Interview Surveys. Persons who reported multiple insurance categories were counted in each category reported, with the exception of Medicaid and the State Children's Health Insurance Program, which were combined into a single category. Denominator data for type of insurance were not available for 1992 through 1994.

In the emergency department, "wound care" can be checked on the patient record form and includes cleaning, debridement, and dressing of burns; repair of lacerations with skin tape or sutures; removal of foreign bodies; excisions; and incision and drainage of wounds provided at the visit. Physician office and outpatient department forms have space to write in ambulatory surgical procedures. ICD-9-CM procedure codes were combined to describe the surgical management of skin and soft tissue infections.

### Statistical Analyses

NAMCS and NHAMCS data were weighted to produce national estimates, and data were combined in 2 groups of 3 years each (1992–1994 and 2001–2003) to provide more reliable estimates. The NAMCS weight includes 4 components: selection probability, nonresponse adjustment, physician-population weighting ratio adjustment, and weight smoothing. Starting with 2001 data, the adjustment for NAMCS physicians who did not provide patient record forms differs from the adjustment used in prior years by taking into account additional characteristics of the physician's practice. Previously, these characteristics were assumed to be the same for physicians who provided information about patient visits and those who did not. The NHAMCS weight includes 3 components: selection probability, nonresponse adjustment, and ratio adjustment to fixed totals. SUDAAN statistical software was used for all statistical analyses (*19*).

The determination of statistical significance was based on the 2-tailed t test (0.05 level of significance). The Bonferroni inequality was used to establish the critical value for statistically significant differences based on the number of possible comparisons within a particular variable (or combination of variables) of interest. Terms relating to differences such as "greater than" or "less than" indicate that the difference is statistically significant. The standard errors used to calculate the 95% confidence intervals (CIs) around the estimates took into account the complex sample designs of the NAMCS and NHAMCS. Estimates based on <30 cases in the sample data did not meet standard of reliability or precision and are indicated in the tables (*20*).

To determine which factors were independently associated with a diagnosis of skin and soft tissue infection, a logistic regression analysis that included all visits was performed. The dependent variable was defined as a diagnosis of skin and soft tissue infection. The model contained the following independent variables: setting type, age, sex, race, expected source of payment, and geographic region.

Before 2003, NAMCS and NHAMCS were exempt from Institutional Review Board review. In February 2003, NAMCS and NHAMCS protocols were approved by CDC's National Center for Health Statistics Research Ethics Review Board. Waivers were granted for the requirements to obtain informed consent of patients and patient authorization for release of patient medical record data by healthcare providers.

## Results

During 2001–2003, a total of 11.6 million annual visits were made to US ambulatory care providers for selected skin and soft tissue infections, representing 1.0% (95% CI 0.9–1.1) of all visits; the visit rate was 410.7 per 10,000 persons. A comparison of the 1992–1994 and 2001–2003 visit rates showed no difference in the rates of overall and physician office visits during the study period; however, the rates for outpatient and emergency department visits increased by 59% and 31%, respectively (Figure). Table 2 and Table 3 show the visit rates and percentage distributions for skin and soft tissue infections according to characteristics of patients, providers, and visits. The proportion of visits made to physician offices decreased from 1992–1994 through 2001–2003, while the proportion of visits to emergency and outpatient departments increased. More than half of all visits (56.2%, 95% CI 52.2–60.2) were initial visits, 33.3% (95% CI 29.6–37.3) were follow-up visits, and 10.5% (95% CI 7.9–13.7) were of unknown episode. No differences in visit rates were found according to sex, race, or metropolitan statistical area status. Rates were higher for children and adolescents 2–18 years of age than for persons >65 years of age and were higher for those residing in the South than those in the Midwest. A greater proportion of visits were made by female patients and a higher proportion occurred in the South than in the other 3 regions. Private insurance was the most frequently recorded expected source of payment, accounting for half of the visits. The visit rates for Medicare and Medicaid patients were higher than for those with private or no insurance. The proportions of visits made by patients eligible for Medicaid (23.9%, 95% CI 19.5–29.0) and patients with no insurance (15.4%, 95% CI 11.8–19.9) were higher for outpatient departments (14.5%, 95% CI 10.7–19.2) than physician offices (4.6%, 95% CI 2.8–7.2). When rates for 1992–1994 were compared with rates for 2001–2003, no differences were observed overall or by age, sex, or race; however, rates increased in metropolitan statistical areas and in the South and decreased in the Midwest.

**Figure:**
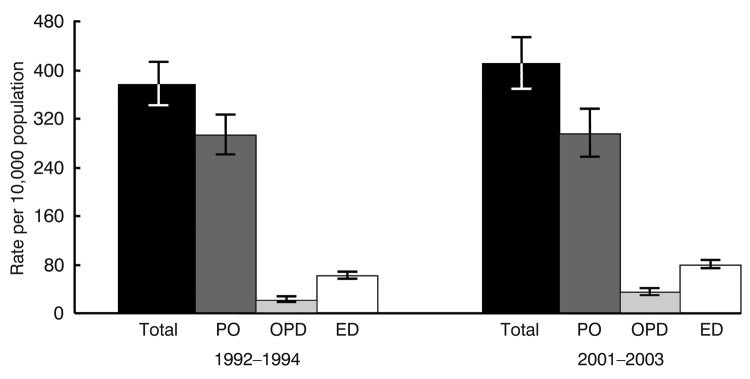
Average rates for annual ambulatory care visits for skin and soft tissue infections, by setting, United States, 1992–1994 and 2001–2003. p<0.001 for rates for outpatient and emergency department visits. PO, physician office; OPD, outpatient department; ED, emergency department. Error bars indicate 95% confidence intervals.

**Table 2:** Average annual number, percent distribution, and rate of ambulatory care visits for selected skin and soft tissue infections, by selected patient, provider, and visit characteristics, United States, 1992–1994*

Characteristic	Visits, in thousands (n)	% Distribution	95% CI		Visits/10,000 persons/y†	95% CI
All visits	9,601	100.0	NA		376.3	340.4–412.3
Setting						
Physician office	7,481	77.9	75.5–80.4		293.2	260.0–326.4
Outpatient department	558	5.8	4.7–7.0		21.9	17.2–26.5
Emergency department	1,562	16.3	14.4–8.2		61.2	55.0–67.5
Patient age, y						
<2	455	4.7	3.4–6.1		569.0	404.8–733.1
2–18	2,050	21.4	18.3–24.4		323.2	265.0–381.4
19–44	3,514	36.6	33.4–39.8		340.8	297.7–384.0
45–64	1,842	19.2	16.5–21.9		371.6	309.9–433.3
>65	1,741	18.1	15.5–20.8		561.0	461.8–660.2
Patient sex						
Female	5,351	55.7	52.0–59.5		408.5	361.2–455.8
Male	4,250	44.3	40.5–48.0		342.4	297.9–386.8
Patient race						
White	8,186	85.3	82.1–88.5		386.6	345.8–427.4
Black or African American	1,158	12.1	9.8–14.3		360.0	289.6–430.3
Other	256‡	2.7‡	0.6–4.7		229.4‡	49.7–409.1
Provider region						
Northeast	2,026	21.1	17.2–25.0		403.4	320.1–486.7
Midwest	2,697	28.1	23.7–32.5		433.3	352.0–514.5
South	2,810	29.3	25.0–33.6		326.9	270.2–383.7§
West	2,068	21.5	17.7–25.4		364.8	291.9–437.7
Provider statistical area						
Metropolitan	7,148	74.5	67.9–81.0		357.9	322.8–393.0§
Nonmetropolitan	2,454	25.6	19.0–32.1		442.8	306.2–579.5
Payment						
Private insurance	4,601	47.9		44.1–51.8	¶	NA
Medicare	1,413	14.7		12.4–17.0	¶	NA
Medicaid or SCHIP	1,409	14.7		12.5–17.0	¶	NA
Uninsured#	1,575	16.4		13.3–19.5	¶	NA
Other**	603	6.3		4.8–7.8	¶	NA

*Numbers may not add to totals due to rounding. CI, confidence interval; NA, not applicable; SCHIP, State Children's Health Insurance Program. †Visit rates for age, sex, race, and region are based on the average of US Census Bureau estimates of the civilian noninstitutional population of the United States, for July 1, 1992, July 1, 1993, and July 1, 1994. ‡Does not meet standard of reliability or precision. §95% CI for 1992–1994 and 2001–2003 overlap, but p<0.05. ¶Denominator data not available. #Includes self-pay, charity, and no charge. **Includes Worker's Compensation, other payment, unknown, and blank.

**Table 3:** Average annual number, percent distribution, and rate of ambulatory care visits for selected skin and soft tissue infections, by selected patient, provider, and visit characteristics, United States, 2001–2003*

Characteristic	Visits, n in thousands	% Distribution		95% CI	Visits/10,000 persons/y†	95% CI
All visits	11,618	100.0		NA	410.7	368.7–452.7
Setting						
Physician office	8,370	72.0		68.8–75.1	295.9	255.9–335.9
Outpatient department	986	8.5		7.1–10.2	34.9	29.1–40.6
Emergency department	2,262	19.5		17.3–21.9	80.0	72.3–87.7
Patient age, y						
<2	576	5.0		3.4–7.3	721.5	434.2–1008.9
2–18	2,292	19.7		16.8–23.1	333.7	272.6–394.7
19–44	3,921	33.7		30.2–37.5	369.6	315.8–423.5
45–64	2,793	24.0		20.4–28.1	422.2	346.7–497.6
>65	2,036	17.5		13.7–22.1	599.2	431.9–766.6
Patient sex						
Female	6,403	55.1		51.5–58.7	442.0	388.2–495.7
Male	5,216	44.9		41.3–48.5	377.9	329.1–426.7
Patient race						
White	9,427	81.1		77.2–84.6	412.1	365.6–458.5
Black or African American	1,635	14.1		11.5–17.1	462.8	362.3–563.3
Other	556	4.8		3.0–7.7	296.1	149.3–442.8
Provider region						
Northeast	2,323	20.0		16.1–24.5	435.3	333.9–536.8
Midwest	2,046	17.6		14.5–21.2	319.3	255.7–382.8
South	4,641	40.0		34.9–45.3	459.7	377.3–542.2‡
West	2,608	22.5		18.7–26.7	404.4	325.0–483.9
Provider statistical area						
Metropolitan	9,775	84.1		79.3–88.0	425.4	374.9–476.0‡
Nonmetropolitan	1,843	15.9		12.0–20.7	347.1	249. 1–445.1
Payment						
Private insurance	5,853	50.4		46.0–54.8	302.8	264.0–341.5
Medicare	2,049	17.6		13.8–22.2	591.2	425.6–756.7
Medicaid or SCHIP	1,889	16.3		13.4–19.7	675.3	530.9–819.7
Uninsured§	990	8.5		6.9–10.4	242.9	192.9–293.0
Other¶	837	7.2		5.6–9.3	#	NA

*Numbers may not add to totals due to rounding. CI, confidence interval; NA, not applicable; SCHIP, State Children's Health Insurance Program. †Visit rates for age, sex, race, and region are based on the average of US Census Bureau estimates of the civilian noninstitutional population of the United States, for July 1, 2001, July 1, 2002, and July 1, 2003; population estimates of metropolitan statistical area status are based on data from the 2001, 2002, and 2003 National Health Interview Survey, National Center for Health Statistics, adjusted to the US Census Bureau definition of core-based statistical areas; and denominator data for expected source of payment are based on the 2001, 2002, and 2003 National Health Interview Survey, National Center for Health Statistics. ‡95% CI for 1992–1994 and 2001–2003 overlap, but p<0.05.§Includes self-pay, charity, and no charge. ¶Includes Worker's Compensation, other payment, unknown, and blank. #Denominator data not available.

Skin and soft tissue infection visits by diagnosis are displayed in Table 1. During 2001–2003, "other cellulitis and abscess" was diagnosed at 53.2% of visits; the visit rate for this diagnosis had increased by 26% since 1992–1994. In contrast, the visit rate for impetigo decreased by 32% during the study period.

Approximately 22.0% (95% CI 19.2–25.0) of visits for skin and soft tissue infection were related to injury. However, the cause of injury is not linked to diagnosis on the patient record form. During 2001–2003, the leading causes of injury were natural and environmental factors (including insect and animal bites) (22.4%, 95% CI 16.7–29.4), being unintentionally cut or pierced by instruments or objects (10.2%, 95% CI 6.7–15.1), and being accidentally struck against or struck by objects or persons (8.4%, 95% CI 5.1–13.5).

In the emergency department, wound care was provided at 41.6% (95% CI 36.7–46.7) of visits for injury-related skin and soft tissue infection. For injury- and illness-related skin and soft tissue infection visits, wound care was provided at 31.3% (95% CI 28.6–34.2) of visits. In physician office and outpatient department settings, procedures related to the surgical management of skin and soft tissue infections were ordered, scheduled, or performed at 9.6% (95% CI 7.3–12.4) of visits.

Antimicrobial drugs were prescribed at 64.6% (95% CI 60.8–68.2) of visits for skin and soft tissue infections during 2001–2003. Between 1992–1994 and 2001–2003, no differences were found in antimicrobial drug prescribing rates overall or for selected therapeutic subclasses, except for cephalosporins, which were prescribed at a higher rate during 2001–2003 and lincosamides/macrolides, which were prescribed at a higher rate during 1992–1994 (Table 4).

**Table 4:** Average annual antimicrobial prescribing rates at ambulatory care visits for selected skin and soft tissue infections, by therapeutic subclass, United States*

Therapeutic subclass†	1992–1994		2001–2003	
	No. prescriptions/10,000 visits/y	95% CI	No. prescriptions/10,000 visits/y	95% CI
All visits	6,899.9	6431.5–7368.3	7,298.6	6870.7–7726.5
Cephalosporins	3,039.3	2,704.5–3374.1‡	3,558.3	3,191.2–3,925.4‡
Penicillins	1,098.7	826.1–1371.3	1,404.2	1,141.0–1,667.4
Lincosamides and macrolides	1,377.7	1,081.9–1673.5	668.7	508.6–828.8
Quinolones	§	NA	646.3	472.8–819.8
Sulfonamides and related compounds, antibacterial agents, miscellaneous	580.8	390.9–770.7	542.2	361.9–722.5
Tetracyclines	134.2	73.6–194.8	258.3	146.8–369.8

*CI, confidence interval; NA, not applicable. †Therapeutic subclass is based on the standard 4-digit drug classification used in the National Drug Code Directory, 1985 and 1995 editions, respectively. ‡1992–1994 95% CI overlaps with 2001–2003 95% CI, but p<0.05. §Does not meet standard of reliability or precision.

During 2001–2003, of all visits for skin and soft tissue infections, 4.0% (95% CI 3.0–5.3) resulted in hospital admission; when limited to visits to the emergency department only, the percentage was 13.6% (95% CI 10.5–14.7). These percentages did not differ from those observed during 1992–1994, which were 3.6% (95% CI 2.6–4.7) and 12.6% (95% CI 2.5–14.7), respectively. For all visits for skin and soft tissue infection, no follow-up was planned for 7.4% (95% CI 5.6–9.6), and referral to another physician was made for 14.4% (95% CI 12.4–16.6).

A multivariate model of factors associated with a skin and soft tissue infection diagnosis for 2001–2003 showed independent associations for the following: emergency department setting, male sex, payment by Medicaid, and residence in the South and West (Table 5). The results from the overall model were significant (p<0.001).

**Table 5:** Factors associated with ambulatory care visits for skin and soft tissue infection

Factor	Adjusted odds ratio (95% confidence interval)
Setting	
Physician office	Referent
Emergency department*	2.0 (1.8–2.4)
Outpatient department	1.2 (1.0–1.4)
Age, y	
<2	0.9 (0.6–1.4)
2–18	1.2 (1.0–1.6)
19–44	1.2 (1.0–1.5)
45–64	Referent
>65	0.7 (0.5–1.0)
Sex	
Female	Referent
Male*	1.2 (1.0–1.4)
Payment	
Private insurance	Referent
Medicaid*	1.4 (1.1–1.8)
Medicare	1.3 (0.9–1.9)
Uninsured	1.2 (0.9–1.5)
Other insurance	1.0 (0.7–1.3)
Region	
Midwest	Referent
Northeast	1.1 (0.9–1.5)
South*	1.3 (1.1–1.7)
West*	1.4 (1.1–1.8)

*p<0.05.

## Discussion

We estimate 11.6 million ambulatory healthcare visits for skin and soft tissue infections possibly due to S. aureus in the United States each year from 2001 through 2003. No change in the overall visit rate for skin and soft tissue infections was found when compared with 1992–1994; however, an increase in these visit rates was observed in hospital emergency and outpatient departments, many for infections coded as cellulitis or abscess. This trend is consistent with findings from a study conducted in a Los Angeles emergency department, where the prevalence of MRSA among patients with skin and soft tissue infections rose from 29% in 2001–2002 to 64% in 2003–2004 (*21*). These data indicate that the number of S. aureus skin and soft tissue infections is substantial and that the emergence of CA-MRSA may affect ambulatory healthcare in the United States. Although we did not identify the causes of the infections, the increase in the rate of visits for cellulitis or abscesses may in part reflect the emergence of CA-MRSA. Many reports of CA-MRSA skin and soft tissue infections have been documented in either closed populations with frequent skin-to-skin contact (*22**–**25*) or emergency department patients and patients admitted through an emergency department (*4**,**8**,**25*). Published reports have indicated that CA-MRSA strains, especially those with the Panton-Valentine leukocidin toxin, are more likely to cause abscesses with a necrotic center that progress rapidly (*6**–**8*). This rapid progression of lesions, frequently described as spider bites (*26**,**27*), may lead persons to seek care in emergency departments rather than physician offices. A possible explanation for the increased visits to outpatient departments but not physician offices is differences in certain patient demographic and medical characteristics (*28**,**29*).

For all settings during 2001–2003, skin and soft tissue infections were independently associated with Medicaid reimbursement relative to private insurance. For all visits made to emergency and outpatient departments in 2003, utilization rates were about 4 times higher for Medicaid recipients than for those with private insurance (*28**,**30*); however, no difference was found for those who visited physician offices (*29*). CA-MRSA might disproportionately affect particular socioeconomic groups who are more likely to seek care in certain settings, which in turn might increase skin and soft tissue infection visit rates to some ambulatory care settings (*5*). However, this finding does not mean that visit rates to other ambulatory care settings will not increase as CA-MRSA continues to emerge.

According to the results of our multivariate analysis, the demographic groups at greatest risk for skin and soft tissue infections likely caused by S. aureus are patients who are male, reside in the South or West, and receive Medicaid. In contrast to the age groups affected by most health conditions, the oldest age groups are at lower risk. Because of the contagious nature of S. aureus strains responsible for skin and soft tissue infection, younger men who have skin-to-skin contact, such as those who play on athletic teams, may be more likely to acquire the infection (*23**,**24*). The association with certain geographic regions may reflect the distribution of demographic groups at highest risk or, alternatively, climate factors (e.g., higher heat and humidity) conducive to skin and soft tissue infections.

As CA-MRSA continues to emerge, monitoring its effect on therapy and whether clinicians are responding appropriately will be helpful. For abscesses, incision and drainage constitute the most important form of primary therapy (*31**,**32*). For >30% of all visits to emergency departments and 10% of visits to outpatient departments and physician offices, wound care, which could include incision and drainage, was provided. Logically, provision of wound care would be higher in emergency departments than in physician offices or outpatient departments, given that patients with more severe infections are generally referred to this setting. However, another explanation of the difference in the 3 settings may be manner of data collection. In the emergency department, wound care is indicated by checking a box, whereas in the other settings the procedure is written in. Write-in items generally have a higher nonresponse rate than check-box items (*33*).

With the continued emergence of CA-MRSA, the clinical management of skin and soft tissue infections has returned to the basic principles of surgical drainage and debulking, wound culture, and the use of older antimicrobial agents other than β-lactams (*34*). However, our results indicate that β-lactam drugs consisting of cephalosporins and penicillins remain the most commonly prescribed therapy for skin and soft tissue infections and that the rate of use of cephalosporins increased over the 12-year study period. A recent study found that for 57% of patients seen in emergency departments for skin and soft tissue infections associated with MRSA, the infecting isolate was resistant to the agent prescribed (*4*). Before the emergence of CA-MRSA, the most appropriate form of antimicrobial therapy for skin and soft tissue infection was β-lactams (assuming penicillins consisted of antistaphylococcal agents). Now clinicians must take into account local and regional rates of CA-MRSA and consider the use of agents such as clindamycin (a lincosamide) or trimethoprim-sulfamethoxazole in the empiric treatment of skin and soft tissue infections (*32*). Periodic monitoring of antimicrobial drug use may be helpful as CA-MRSA continues to emerge.

Our study has several limitations. Most important is the fact that these ICD-9-CM codes have not been validated as a method for tracking skin and soft tissue infections likely to be caused by S. aureus, much less infections caused by CA-MRSA. In addition, whether the baseline risks for skin and soft tissue infections were similar between the 2 periods studied is unknown. Rates for conditions such as diabetes, peripheral vascular disease, traumatic injuries, and homelessness might differ for the 2 periods, which would obscure any actual increase in skin infections due to MRSA. Although 3 years of data were combined, some estimates were not presented because they were unreliable, and some estimates for diagnoses, drugs, and procedures were aggregated into broader categories to attain reliability. Because the design of NAMCS and NHAMCS does not allow for patient follow-up, some cases may have been counted multiple times. Because diagnosis cannot be associated with a particular drug, we could only assume that the antimicrobial drug listed was prescribed for the skin or soft tissue infection diagnosis recorded at the same visit. Procedure data are collected differently in emergency departments than in physician offices and hospital outpatient departments and, therefore, are not comparable. Skin and soft tissue infections misdiagnosed as spider bites may not have been captured in NAMCS or NHAMCS. We found that the rate for all visits assigned a cause-of-injury E-code of 905.1 (venomous spiders) increased significantly, from 2.7 per 10,000 persons (95% CI 1.5–3.9) during 1992–1994 to 8.4 (95% CI 4.9–11.9) during 2001–2003.

In conclusion, we found that the number of skin and soft tissue infections is increasing in hospital emergency and outpatient departments; this increase may reflect the emergence of CA-MRSA. However, despite these increases, changes in the therapeutic approach to these infections are not apparent. These findings may serve as a baseline for future analyses to track the continued emergence and effect of CA-MRSA on ambulatory healthcare and to monitor how clinicians adapt and treat these patients.
